# High-capacity adsorbents from stainless steel slag for the control of dye pollutants in water

**DOI:** 10.1007/s11356-020-12174-0

**Published:** 2021-01-02

**Authors:** Lorenzo Plaza, Marta Castellote, Roman Nevshupa, Eva Jimenez-Relinque

**Affiliations:** Spanish National Research Council, Eduardo Torroja Institute of Construction Science (IETcc-CSIC), C/ Serrano Galvache, 4, 28033 Madrid, Spain

**Keywords:** Stainless steel slag, Adsorbent materials, BET surface area, Synthesis, Methylene blue, Water pollution control, Dye

## Abstract

Adsorbent materials for the control of dye pollutants in water were synthetized from stainless steel slag (SSS) using different acid-base treatments. Using HCl (SS-Cl) and HNO_3_ (SS-NO_3_) produced high-capacity adsorbents, with BET areas of 232 m^2^/g and 110 m^2^/g respectively. Specifically, the SS-Cl had a structure of amorphous silica sponge. Treatment with H_2_SO_4_ (SS-SO_4_) did not enhance the adsorption capabilities with respect to the raw sample (SSS). Activated carbon (AC) was also tested as reference. The materials were characterized by X-ray diffraction (XRD), X-ray fluorescence (XRF), N_2_ adsorption-desorption isotherms, scanning electron microscopy (SEM), energy dispersive X-ray analysis (EDX) zeta potential, and infrared spectroscopy (FTIR). Batch adsorption experiments with methylene blue (MB) showed that the maximum sorption capacities were 9.35 mg/g and 8.97 mg/g for SS-Cl and SS-NO_3_ at 240 h, respectively. These values, even at slower rate, were close to the adsorption capacity of the AC (9.72 mg/g). This behavior has been attributed to the high porosity in the range of nanopores (0.6–300 nm) and the high-surface area for both samples. Preferential involvement of certain functional groups in the adsorption of dye ions on their surface indicative of chemisorption has been found. Although optimization, repeatability, and reproducibility of the process and environmental assessment have to be done before practical applications, these preliminary results indicate that application of these cost-effective adsorbents from raw SSS may be used in water pollution treatment and contribute to the sustainable development of the steel manufacturing industry.

## Introduction

Steel manufacturing produces a significant amount of different slags (10–15% by weight of the produced steel) (Proctor et al. 2000). It can be categorized as carbon steel slag and stainless steel slag (SSS) according to the type of steel, and as pretreatment slag, basic oxygen furnace slag (BOFS), electrical arc furnace slag (EAFS), ladle refining slag (LFS), and casting residue according to the steelmaking process (Manchisi et al. 2020; Yi et al. 2012). The steel slag mainly consists of SiO_2,_ CaO, Fe_2_O_3_, FeO, Al_2_O_3_, MgO, MnO, and P_2_O_5_. The composition of steelmaking slags varies greatly from batch to batch, depending on the type of steel produced (e.g., carbon steel vs. stainless steels), the quality of starting raw materials and pretreatment process (Yi et al. 2012).

Based on steel production data of EU for the year 2018, around 16 million tons of steel slags are generated annually, 53% of which made up by BOFS, followed by EAFS (35%) and LFS (12%) (Euroslag 2018). For a long time, slags have been considered a problem, due to the high costs associated with their subsequent treatments, storage and disposal. However, the perception towards this waste is progressively changing and improving its reuse. Making reference again to EU data for the year 2018, 73% of these by-products are typically reused as artificial aggregates in road construction and cement production, but 13% are still landfilled, and 14% appear as internal storage (Euroslag 2018). To improve the current management practices for steel slags, recent studies have been focused on the development of recycling technologies, discovering the great potential of this type of waste as a precursor to synthesize products with high-added value. The valorization of such large amounts of steel slag are of great importance, both for the sustainability of the metallurgical industry and for the environment. One of the approaches to convert slag into a product with new potentialities is focused on exploring the effect of accelerated carbonation for storing point-source emissions of CO_2_ (Baciocchi et al. 2010; Bonenfant et al. 2008; Huijgen et al. 2005; Johnson 2000; Kodama et al. 2008; Lekakh et al. 2008; Santos et al. 2013). This conversion is accompanied by improving the mechanical properties, significant reduction in basicity, and stabilization of heavy metal leaching (Garcia-Blas et al. 2019; Johnson et al. 2003; Van Gerven et al. 2005). Black furnace slags (BFS) are used widely as supplementary materials in cements, having the cements type CEM V/B until a 49% of substitution of clinker by slags (Roy 1982), increasing in a big deal its durability (Castellote and Andrade 2006; Osborne 1999). However, the use of SSS has been constrained mainly due to the presence of heavy metals (Gomes and Pinto 2006), even though it can also be used as supplementary cementitious material (Shi 2004). The steel slag has been also used to remove metals (Repo et al. 2015; Zhou and Haynes 2011), phosphate, and dyes (Ahmed and Ahmaruzzaman 2016; Cheng et al. 2018; Xue et al. 2009) from contaminated water. However, the adsorption capacity of steel slag is relatively low mainly due to its low-specific surface area. Steel slag presents a mesoporous and mainly an amorphous structure with a typically low-specific surface area of less than 10 m^2^/g (Yi et al. 2012). This challenge notwithstanding, the efficacy of slag-based adsorbents can be significantly enhanced through purposeful activation to increase the specific surface area and density of adsorption sites on the surfaces of adsorbent particle. In addition, it is easy to separate from water due to its high density (3.3–3.6 g/cm^3^). Different works are currently under way to find alternative conversion routes to develop sustainable slag-based materials for contaminated water treatment. Owing to CaO-SiO_2_-MgO-Al_2_O_3_ main system composition of steel slags, it can be used as a precursor for preparing a high-surface area calcium silicate hydrate, zeolite, or layered double hydroxide structures (Chiang et al. 2014; Kuwahara et al. 2009, 2010; Kuwahara et al. 2013). On this respect, activation routes based on the combination of acid-base treatments allow the adsorption capacities to be improved effectively. In Kuwahara et al. (2009), BFS is converted to a hydroxyapatite-zeolite compound using aqueous solutions of H_3_PO_4_ and NaOH. In Kuwahara et al. (2010) and Kuwahara et al. (2013), a double-layered hydroxide based on Ca and zeolite structures through two-stage dissolution procedures of coprecipitation using aqueous solutions of HCl and NaOH, respectively, was synthesized. In Chiang et al. (2014), a three-step process to transform BFS into two valuable products in parallel was developed: precipitated calcium carbonate and zeolite material. The materials synthesized in these works showed good adsorption properties allowing their use as adsorbents/solid ion exchangers for the treatment of wastewater and the elimination of dangerous substances in water such as heavy metal, dyes, arsenic, and phosphate ions. Furthermore, volatile organic compounds (VOCs) in the air are as adsorbents. Kuwahara et al. (2020) synthesized a mesoporous silica-calcium oxide compound from BFS by a hydrothermal dissolution process using formic acid as the solution. The synthesized composite material had a CO_2_ adsorption value of 18.8%, and could be used for at least 10 cycles.

These precedents show that steel slags have great potential as a precursor to synthesize products with adsorbent characteristics. Efficient use of these low-cost sorbent materials can become an alternative technology for environmental remediation, and ultimately would lead to reduced waste generation. However, to maximize the chemical and economic potential of this type of waste, technologically viable conversion processes and implementable to the broadest range of slag type are needed. Most of the previous works were focused fundamentally on BFS-type slag residues without SSS ever being studied. The stainless steel market is witnessing a surge in demand, owing to the growth of end-user industries such as construction, automotive industry, and hygiene applications. Therefore, improving the utilization rate of stainless steel slag is an imperative way for the steel making industry to a sustainable development.

On the other hand, the use of dyes in many industries such as paint, textile, tannery, paper, leather, rubber, cosmetics, and plastics has resulted in the release of large amounts of colored toxic effluent and contaminated surface groundwater (Forgacs et al. 2004). About 10,000 types of commercial dyes are commonly used with around 7 × 10^5^ t of dyestuffs produced annually. Around 2% of them are released in effluents. The dyes significantly not only compromise the esthetic quality of water bodies but also may promote mutagenicity and carcinogenicity (Forgacs et al. 2004). Adsorption method has been widely accepted as one of the most effective dyes removal water treatment because it is rapid, effective, producing nontoxic by-product, and simple in operation design (Raval et al. 2016). Thus, the study and application of steel slag as low-cost adsorbent for the elimination of dye from contaminated wastewater is a very important issue that should be addressed.

In this work, the production and application of modified stainless steel slags (SSS) based adsorbents for the efficient removal of a cationic organic dye methylene blue (MB) from wastewater has been demonstrated. Three acid-base treatments were used to improve the adsorption property of ball-milled SSS for the first time. Physico-chemical characterizations were conducted to obtain the changes in properties of the steel slag before and after the modification. Comparative of reported main textural properties and adsorption capacities of the analyzed samples with the literature data of various modified slag materials and of a selected number of commercial adsorbents are also included.

## Experimental section

### Acid-base treatments of raw stainless steel slag

**Fig. 1:**
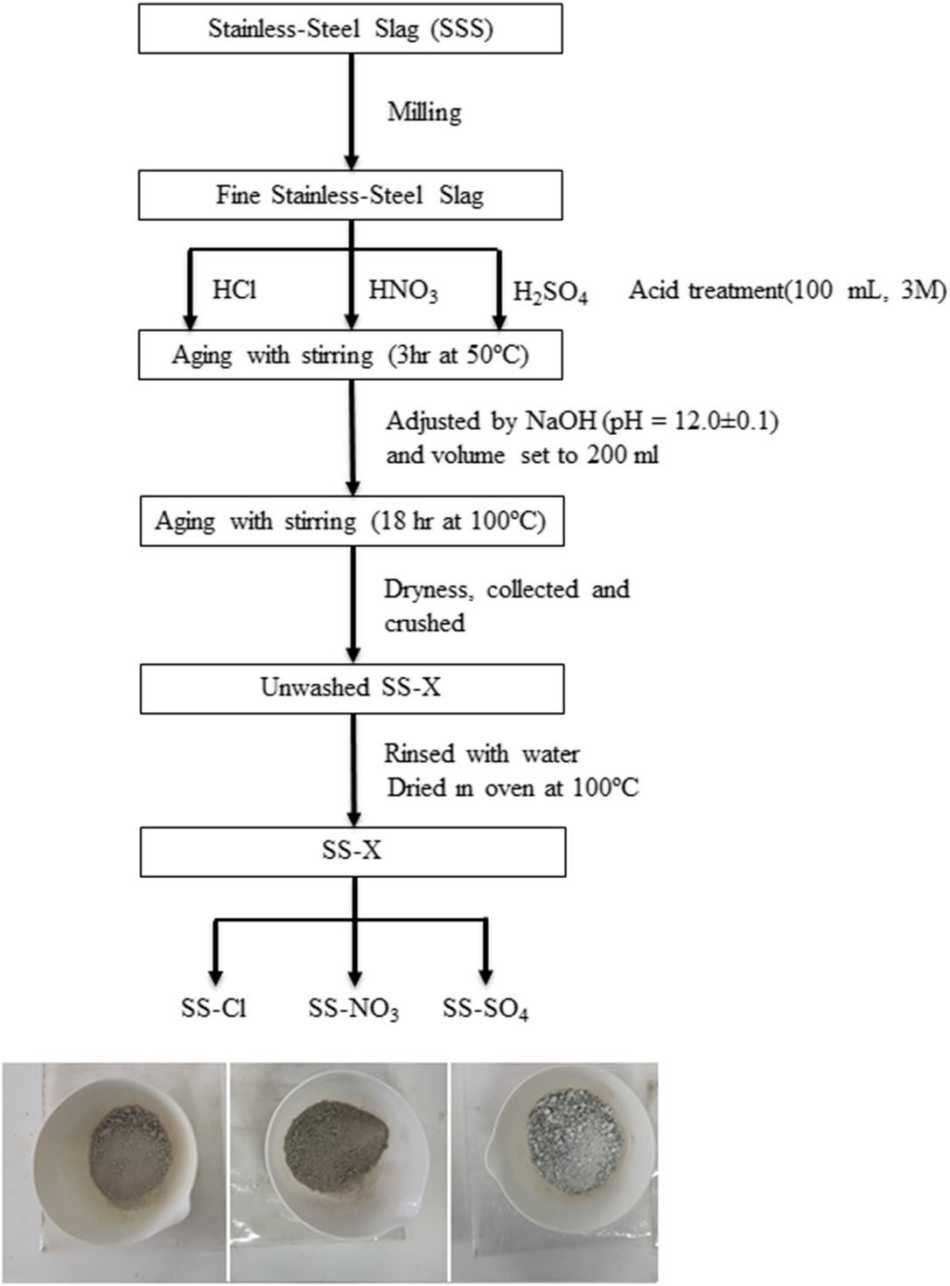
Flow chart of acid-base treatments of the raw stainless steel slag (SSS) and image of the resulting treated slags. From left to right SS-Cl, SS-NO_3_, SS-SO_4_

The SSS used in this study was provided by Acerinox Europa, S.A.U., Spain. Three acid-base treatments were explored using the following acids: HCl, HNO_3_, and H_2_SO_4_ and the procedure shown in Fig. 1. The materials obtained were denoted according to the acid employed: SS-X, X=Cl, NO_3_, SO_4_. Before the treatments, the SSS was milled in a Micro Fine Mill Grinder at 5000 rpm and sieved using a 120-μm mesh. A weight of 5.0 g of the milled SSS was dissolved in 100 ml of 3M acid solution. In order to avoid gelation, the solution was strongly stirred for 3 h at 50 °C. After that, 4M NaOH aqueous solution was added dropwise until pH reached 12 ± 0.1. The volume was set to 200 ml with water followed by stirring at 100 °C until dryness is reached after 18 h. The formed solid was crushed, thoroughly rinsed with water, and dried in an oven at 100 °C overnight. The yield of the process was 5.4 g, 6.5 g, and 8.2 g for SS-Cl, SS-NO_3_, and SS-SO_4_, respectively (Fig. 1).

### Characterization of the samples

The X-ray diffraction patterns were obtained using a Bruker D8 Advance diffractometer with CuKα radiation (40 kV, 30 mA). The elemental analysis of the samples were performed by X-ray fluorescence (XRF) using a X-ray Fluorescence Bruker S8 Tiger. The surface morphology and the composition analysis of samples were carried out by scanning electron microscope (SEM, JSM-6700F system) equipped with an energy dispersive X-ray fluorescence spectrometer (EDX), by which the optical analysis and elemental mapping were performed. A detailed analysis of the structure of the samples was performed by infrared spectroscopy.

Nitrogen adsorption–desorption isotherms were obtained using a conventional volumetric apparatus (ASAP2010) at 77 K. The linear BET plots (SBET) were used to determine the equivalent surface area and the pore size distribution of samples was calculated by BJH method. Zeta potential analysis was conducted on samples dispersed in deionized water (10 mg/50 ml) using a Zetasizer (Zeta-meter 3.0+). Activated carbon (AC) was also tested to compare the results with a reference. AC sample was also milled in a Micro Fine Mill Grinder Culatti at 5000 rpm and sieved using a 120-μm mesh.

### Methylene blue adsorption tests

Adsorption tests were performed using methylene blue (MB), which was chosen because of the neat charge at pH solution (pH = 8.0), and benchmarked against untreated raw SSS and AC. Prior to tests, all samples were preheated at 363 K to remove physisorbed H_2_O.

In a first test, the adsorption performance was evaluated by mixing the solid sample (30 mg) with MB solution (10 ml, 30 ppm) in sealed glass vessels placed on a rotator for up to 240 h at 50 rpm. At predetermined time intervals, aliquots were taken and subsequently centrifuged to remove the solid sample. The decrease in absorbance of the supernatant solution was determined using Shimadzu UV-Vis spectroscopy. MB concentration was determined at 668 nm using a calibration line in the range 0–10 mg/l. The adsorbance efficiency (%) for MB was determined from the following formula:

1 $$ \left(\%\right)=\frac{\left({C}_0-{C}_t\right)}{Co}\times 100 $$

The amount of MB adsorbed, *q* (mg/g), was obtained as follows (Eq. 2):

2 $$ q=\frac{\left({C}_0-{C}_t\right)\times V}{m} $$

where *C*_*0*_ and *C*_*t*_ are the concentrations of MB (mg/l) at times 0 and *t* respectively, *V* is the volume of the solution (l), and *m* is the weight of the solid sample (g).

In the second test, the samples (50 mg) were saturated using a concentrated dissolution of the MB dye (250 ml, 70 ppm) in sealed containers placed on a rotator for 2 weeks (336 h) at 50 rpm. After filtration and drying at 100 °C overnight, the solid samples were analyzed by FTIR (Thermo Scientific Nicolet 6700 Spectrometer) to identify the functional group that participate in the adsorption of dye ions on the surface.

## Results and discussion

### Characterization

**Fig. 2:**
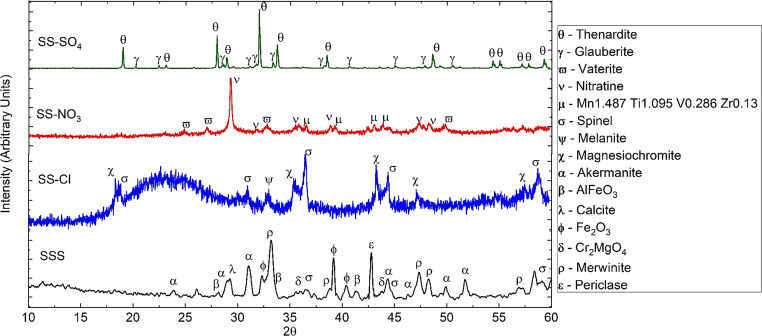
XRD patterns of raw SSS and treated samples

Figure 2 shows the XRD patterns of the treated samples (SS-X), together with the raw SSS material. SSS exhibits diffraction peaks corresponding to mineral phases as akermanite (Ca_2_Si_2_MgO_7_), merwinite (Ca_3_Si_2_MgO_8_), calcite (CaCO_3_), periclase (MgO), and some metallic mixtures (AlFeO_3_, Fe_2_O_3_, and CrMgO_4_ and Mg-Al-Cr spinel structure). The diffraction patterns of the treated samples are quite different with respect to the SSS sample. The first observation is that there are less phases present in the treated samples. Some of the phases in SSS have disappeared, as a consequence of the strong acid treatments and some new phases are present. The sample treated with HCl (SS-Cl) presents high amorphous character with low-defined crystalline phases identified as magnesiochromite (MgCr_2_O_4_), melanite (Ca_3_(Fe^3+^)_2_(SiO_4_)_3_), and Mg-Al-Fe-Cr cubic spinel structure. SS-NO_3_ and SS-SO_4_, did not exhibit amorphous structure, being the major crystalline phase identified in SS-NO_3_, nitratine (NaNO_3_), related with the interaction of the correspondent acid treatment and subsequent addition of NaOH. Vaterite (polymorph of CaCO_3_) and a mixture structure of Mn-Ti-V-Zr could be also observed. The H_2_SO_4_-treated samples (SS-SO_4_) presented thenardite (Na_2_SO_4_) and glauberite (Na_2_Ca(SO_4_)_2_), which stemmed from the treatments.

**Fig. 3:**
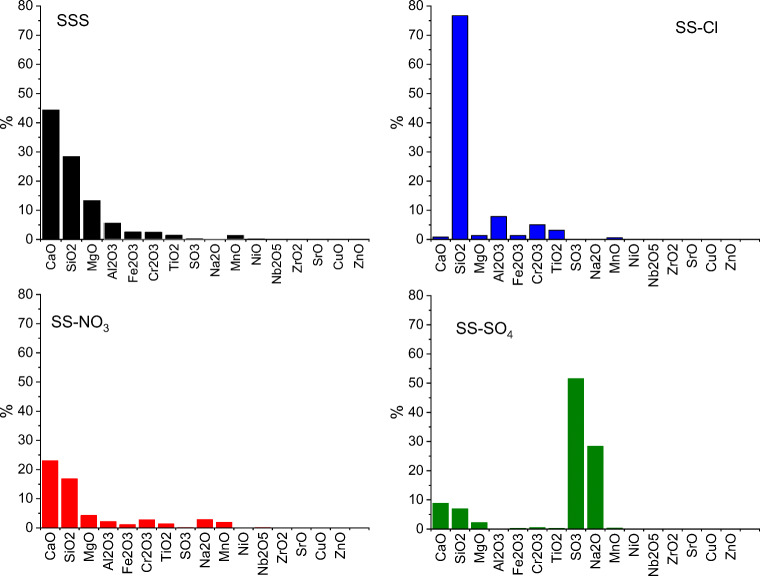
Elemental analysis composition of raw SSS and treated samples

The elemental composition of the treated samples (SS-X) and the raw SSS samples are shown in Fig. 3. The main components of SSS were CaO, SiO_2_, MgO, and Al_2_O_3_ and small amounts of transition metals such as Fe, Cr, Ti, and Mn. The composition was in agreement with the XRD results. The SS-Cl samples consisted mainly of SiO_2_ in amorphous form (~ 77%). The fact that Ca vanished during the treatment was attributed to formation of soluble CaCl_2_, which was removed by washing. The subsequent addition of excess of NaOH may lead to precipitation of Mg- and Si-containing components, because of their insolubility in alkali solution (Kuwahara et al. 2009). The treatments with HNO_3_ (SS-NO_3_) and H_2_SO_4_ (SS-SO_4_) caused decrease of both CaO and SiO_2_ and formation of other compounds, which did not leach during washing. We suppose that insoluble nitrates were formed during HNO_3_ treatments since nitrogen was not detected by XRF. In the H_2_SO_4_ treatment, SO_3_ and Na_2_O could be formed due to fixation of Na in the form of insoluble sulfates. This result is in good agreement with the above XRD identifications.

**Fig. 4:**
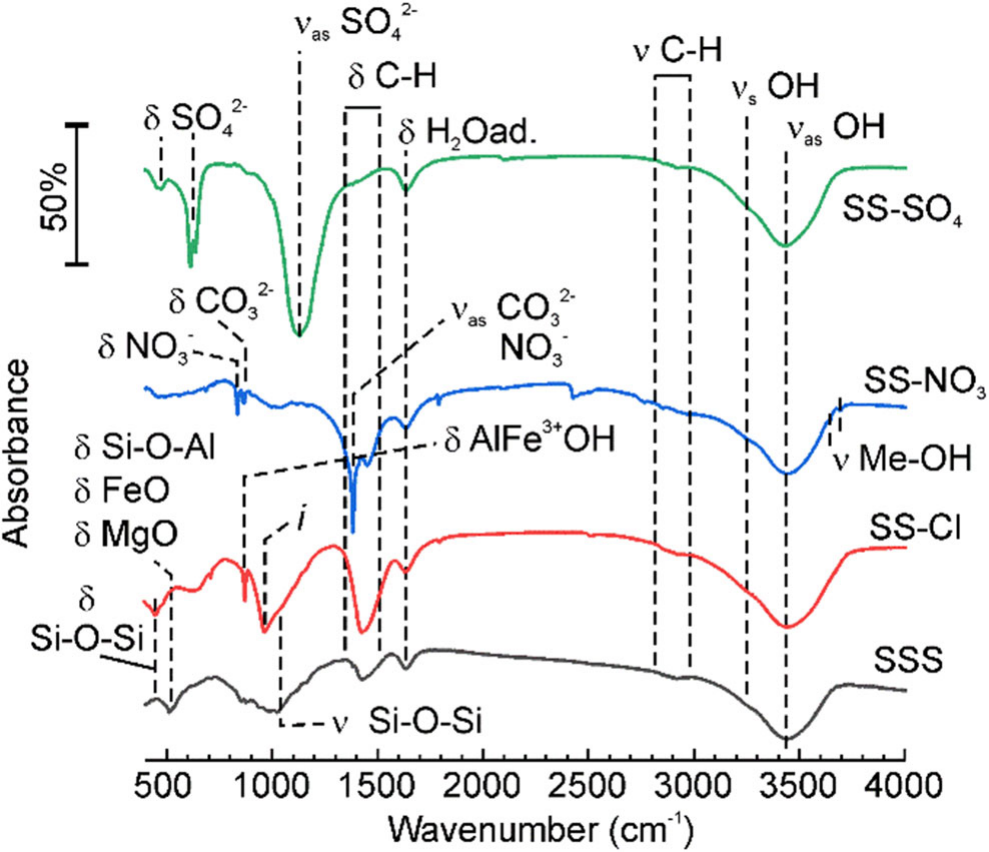
FTIR spectra of the raw (SSS) and treated samples (SS-Cl, SS-NO_3_, and SS-SO_4_)

The FTIR results (Fig. 4) showed good correlation with the compositional analysis. The increase in Si concentration after HCl treatment corresponded to the increase of bands of deformation vibration of Si-O-Si. Furthermore, the band at 512 cm^−1^, which was ascribed to vibrations of various metal-oxygen bonds such as FeO, MgO, and also Si-O-Al (Madejová et al. 2017), vanished after HCl treatment, but the band at 874 cm^−1^ appeared. The latter could be associated with AlFe^3+^OH (Madejová et al. 2017) and various C=C and C=O bonds (Socrates 1994). The wide band at 964 cm^−1^ can be due to superposition of various vibration bands of surface methyl groups, C-O-C and C=C. This is in line with the general increase of the bands corresponding to sp^2^ and sp^3^ hydrocarbons (Rusanov et al. 2015) on the SS-Cl. The SS-NO_3_ samples showed significant contribution from adsorbed CO_2_ and carbonate bands (Falk and Miller 1992; Kloprogge et al. 2002) that agrees observations of vaterite (calcium carbonate) in XRD spectrum of SS-NO_3_. The band of low intensity at 835 cm^−1^ could correspond to nitrates (Kloprogge et al. 2002). In the spectrum of the SS-SO_4_, the bands of sulfate were dominating (Das et al. 2014; Kloprogge et al. 2002; Pu et al. 2006; Wang et al. 2017), in agreement with the FRX data. In addition, all the spectra showed the bands of bonded H_2_O (Soria et al. 2007).

**Fig. 5:**
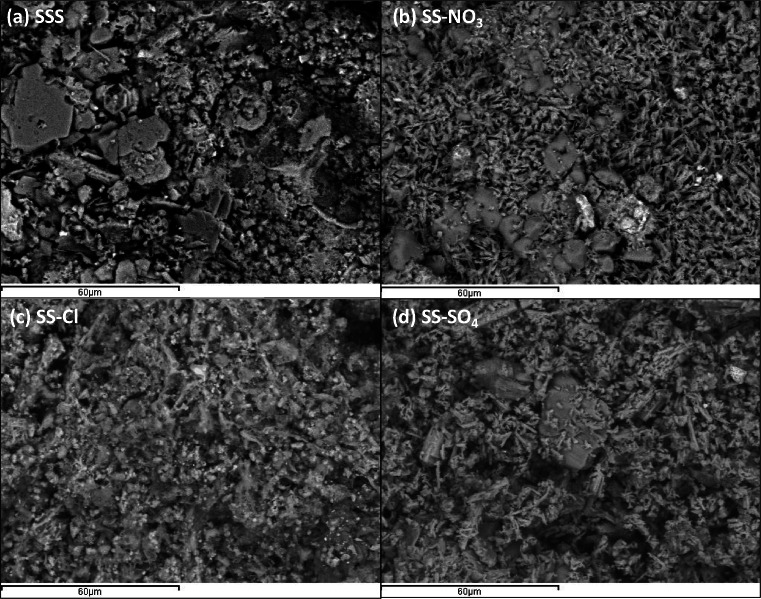
SEM images at × 1000 magnification of **a** SSS, **b** SS-NO_3_, **c** SS-Cl, and **d** SS-SO_4_

The SEM images of raw and treated samples at × 1000 magnification are shown in Fig. 5 (a–d). All the samples had heterogeneous structure with large clusters and small detrital grains. For all treatments, the sample morphology significantly differed from the original SSS. Among the treated samples, SS-NO_3_ and SS-SO_4_, in agreement with the XRD, present nitrate and sulfate crystalline formations respectively distributed all around the samples with more opened macroporosity than in the case of SS-Cl, where the most amorphous character can be seen, exhibiting a more refined apparent porosity.

**Fig. 6:**
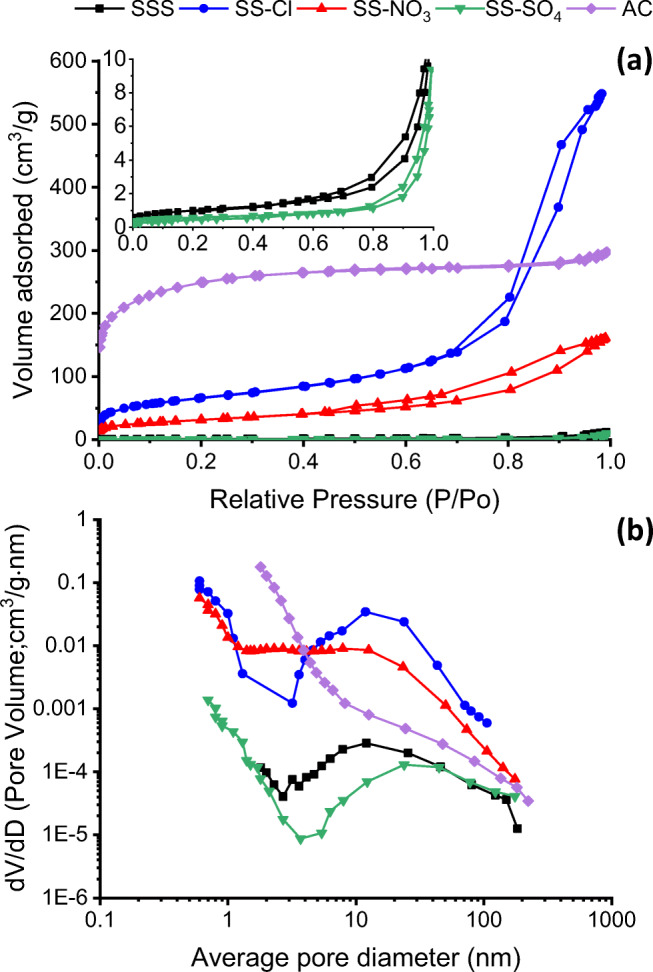
**a** N_2_ adsorption-desorption isotherms and **b** differential pore size distribution in the range of nanopores

Figure 6a shows the N_2_ adsorption-desorption isotherm of all samples and the corresponding pore size distribution calculated according to Barrett-Joyner-Halenda (BJH) equation. The AC showed a reversible type I isotherm with steep uptake at very low *p*/*p*_*0*_ due to narrow micropores and relatively small external surfaces (Thommes et al. 2015). The SSS and the SS-SO_4_ exhibited the isotherms similar to type III with relatively weak adsorbent-adsorbate interactions. In turn, the isotherms for the SS-NO_3_ and SS-Cl were type II, i.e., physisorption of gases on nonporous or macroporous adsorbents. All the treated samples showed a small hysteresis loop of type H3, which is characteristic for non-rigid aggregates of plate-like particles or for macropore network which is incompletely filled with pore condensate.

Figure 6b gives the differential pore size distribution in the range of nanopores (0.6–300 nm). AC sample presents its maximum amount of pores at the lower size analyzed, showing a continuously diminishing trend as the pore diameter increases. SS-Cl, from its maximum at smaller sizes, diminishes to reach another maximum at around 10 nm, being the sample with maximum porosity in this range. SS-NO_3_ presents a stable amount of pores in the range 1–10 nm, to diminish afterwards, having less amount of pores in this range with smaller mean diameter. SS and SS-SO_4_ have porosity an order of magnitude smaller than SS-NO_3_ porosity with higher mean pore diameter.

In agreement with the physisorption isotherms, the SSS and the SS-SO_4_ had lower BET area (3.47 m^2^/g and 1.97 m^2^/g respectively), and low porosity with large pore diameter. The average pore diameter decreased, while pore volume increased after HCl (SS-Cl) and HNO_3_ (SS-NO_3_) treatments. The BET surface area of SS-Cl and SS-NO_3_ are more than 67 and 32 times greater respectively than that of the un-modified SSS, which may imply a higher adsorption capacity. The reference activated carbon (AC) sample had the highest BET surface area (813 m^2^/g), but the order of magnitude is the same that SS-Cl and SS-NO_3_ as-prepared samples. According to these results, the treatment using H_2_SO_4_ yielded the worst results among all. Table 2 lists the values of the main textural properties of the tested samples.

The pH, conductivity, and Z-potential of the samples are given in Table 1. All treated samples maintained the basic character and negative surface charge as the SSS. The decreasing order of negative charge was the following AC > SS-SO_4_ > SS-Cl > SSS > SS-NO_3_, whereas the conductivity increased as follows AC < SSS < SS-Cl < SS-NO_3_ < <SS-SO_4_. This ranking can be due to the existence of different amount of soluble components, mainly in SS-NO_3_ and SS-SO_4_, as previously identified by XRD.

**Table 1:** PH, conductivity and *Z*-potential of analyzed samples

Sample	pH	Conductivity (μS/cm)	*ζ*-potential (mV)
SSS	8.3	32.7	− 19.14
SS-Cl	8.7	65.3	− 22.30
SS-NO_3_	8.0	96.2	− 14.71
SS-SO_4_	7.3	360	− 35.15
AC	6.3	16.1	− 50.15

### Adsorption tests

**Fig. 7:**
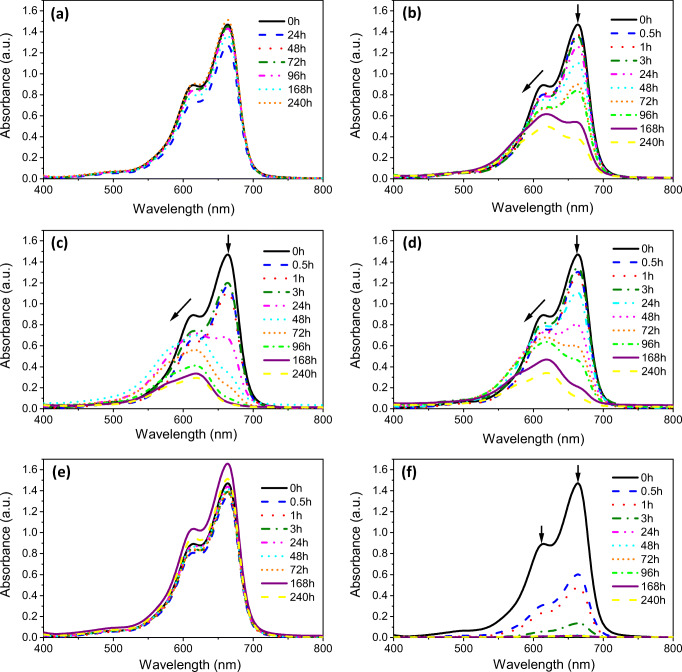
Time evolution of absorption spectra of the MB solutions for **a** blank, **b** SSS, **c** SS-Cl, **d** SS-NO_3_, **e** SS-SO_4_, and **f** AC

**Fig. 8:**
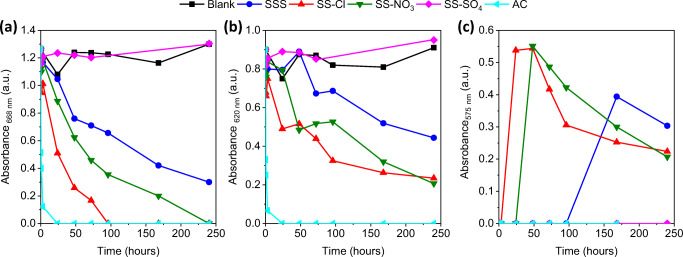
Time profiles of absorbance of MB solution measured at **a** monomer, **b** dimer, and **c** trimer

The adsorption spectra of the MB solutions during the adsorption tests of the samples at different times are shown in Fig. 7 (a–f). At the beginning, the spectra showed two strong MB peaks with the maximum absorption at ~ 668 nm and a small shoulder at ~ 620 nm that was ascribed to the characteristic bands of MB monomers and dimers in solution, respectively (Yuan et al. 2019). In agreement with the low BET area, no MB adsorption happened on the SS-SO_4_, but the dye adsorbed on the AC quickly. For the SSS, SS-Cl, and SS-NO_3_, both monomer and dimer bands decreased during the test, while a broad band below 609 nm appeared. This band can be considered an indication of MB molecular aggregation on the samples surface, which can be assigned to the trimer structures (Horváth et al. 2014; Karaca et al. 2008; Yuan et al. 2019). To understand this behavior better, the adsorption spectra were fitted by the peaks corresponding to each MB form (monomer, dimer, and trimer) as shown in Fig. 8 (a–c). The absorbance at 620 nm, corresponding to dimer structures, did not change or even increased at the beginning of the tests for SSS, SS-Cl, and SS-NO_3._ The trimer band at 575 nm reached maximum in the middle of the test for SS-Cl and SS-NO_3_ and in the second half of the test for SSS. These structures might result from the steric hindrance and a lower diffusion rate in comparison with MB monomers. However, at longer adsorption duration, the agglomerate structures of MB may overcome diffusion resistance between the aqueous and solid phases.

**Fig. 9:**
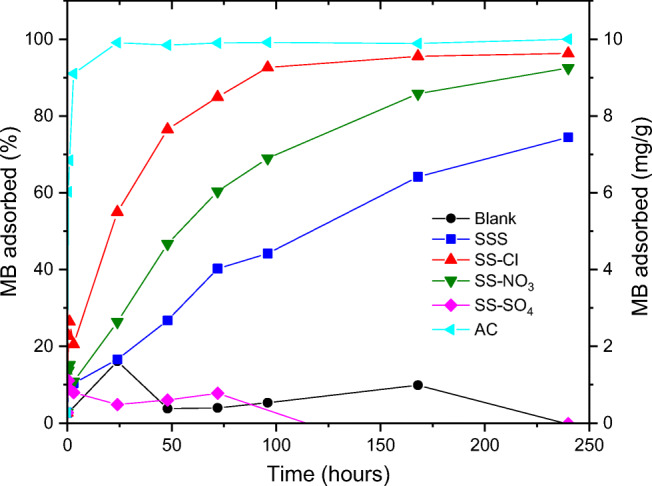
Percentage and amount adsorbed of MB of all samples during the time of adsorption test

**Fig. 10:**
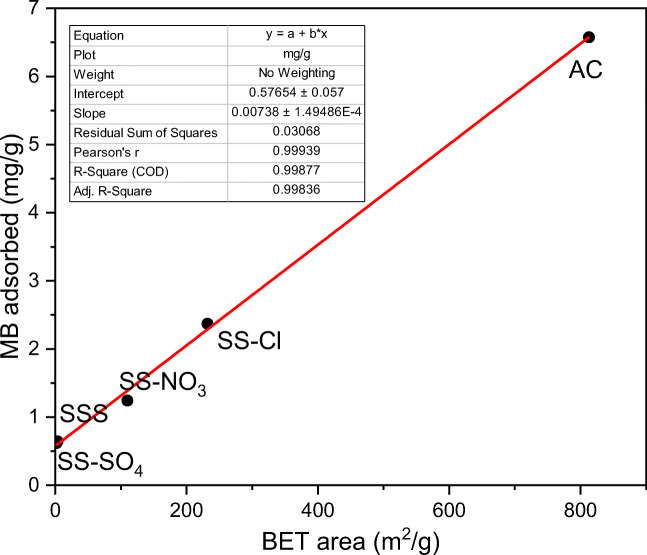
Relationship between BET Surface area and amount of MB adsorbed per gram of sample at 1 h of test

The adsorption percentages and amount of MB adsorbed per gram of sample were calculated from the monomer band at 668 nm (Fig. 9). The adsorption of MB on AC completed during the first 24 h reaching the equilibrium at the maximum 9.72 mg/g. This is due to the high *negative surface charge*, ordered microporous structure and large surface area. The SS-Cl and SS-NO_3_ also showed significate adsorption capability compared to the SSS. The enhanced adsorption capability of these samples is related to the improvement of BET area and pore volume parameters, thus providing more adsorption sites for MB removal. Although in the beginning the adsorption capacity was lower than for AC that could be related to the negative impact of the MB agglomerations at the surface, the SS-Cl and the SS-NO_3_ reached 96% and 93%, respectively (9.35 mg/g and 8.97 mg/g respectively, in absolute value) after 240 h. The maximum adsorption for SSS was 74% (7.17 mg/g). AC and SS-Cl reach the steady adsorption state during the test, which did not occur in the case of SSS and SS-NO_3_. SS-SO_4_ did not presented significant adsorption of MB, which was attributed to lower BET area than SSS. The good linear relationship between the adsorption capacity and BET surface area (Fig. 10) (*R*^2^ = 0.99) demonstrated that the amount of available surface sites is the critical parameter, which governs the adsorption capacity of the samples. The zeta potential should not have significant relevance concerning the adsorption capacity.

**Fig. 11:**
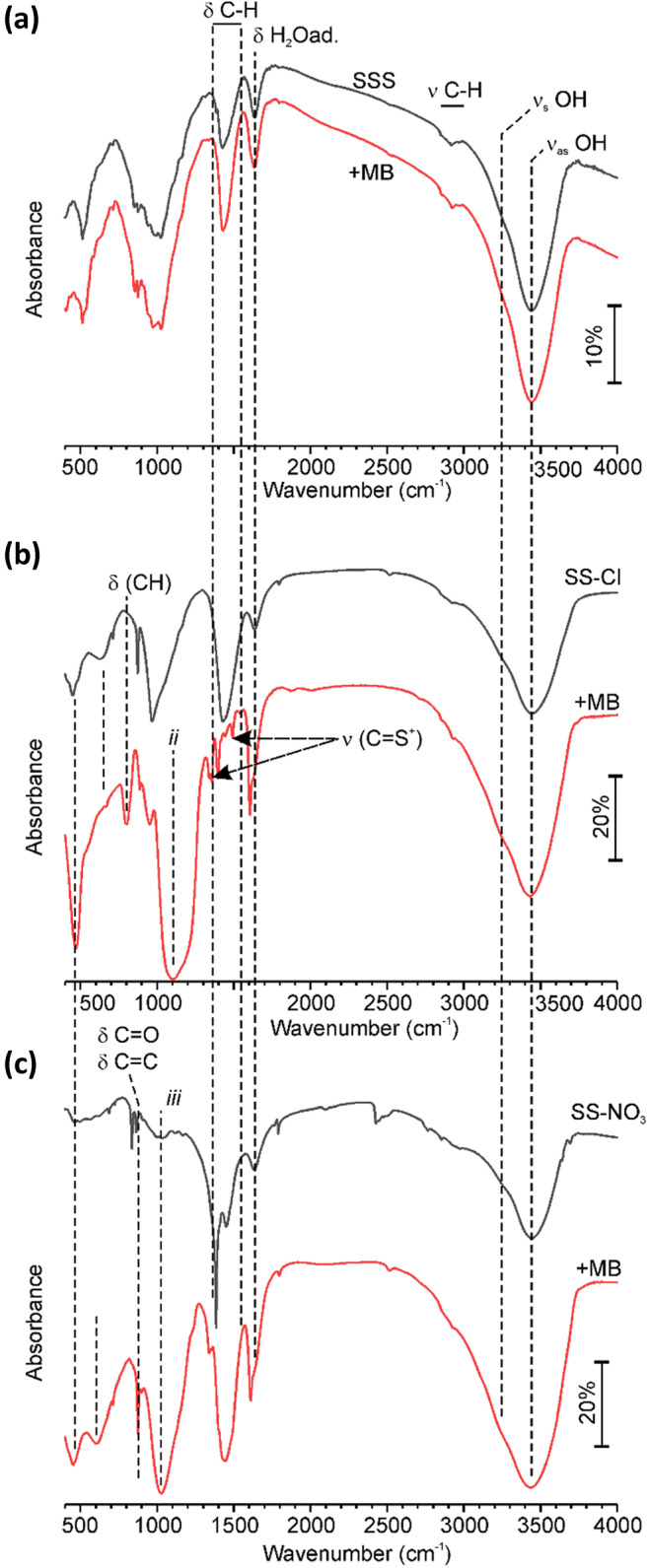
FTIR spectra of various samples before and after MB adsorption: **a** SSS; **b** SS-Cl; **c** SS-NO_3_

To provide further insight into MB adsorption on various samples FTIR analysis was carried out after dye adsorption on a second adsorption test (250 ml, 70 ppm, 336 h) (Fig. 11). The data are shown only for the samples, which had considerable adsorption capacity.

While for SSS, MB adsorption did not have any notable effect suggesting that the adsorption of dye molecules on SSS is through weak electrostatic interaction or van der Waals forces. Notwithstanding, MB adsorption on both SS-Cl and SS-NO_3_ caused significant variation of the FTIR spectra and these variations were different between the samples. In the spectrum of the SS-Cl, the initial broad band of CH deformation vibration vanished and it was substituted by several weak and narrow bands identical to those of neat MB, which were ascribed to CH and C=S^+^ (Ovchinnikov et al. 2016). However, the FTIR spectrum is not a superposition of the spectra of the substrate and neat MB since other MB bands were missing, while new bands appeared. The new wide and strong band centered at 1100 cm^−1^ could be due to the contributions of amine, CO, and tertiary methyl or methyl-Al bonds (Socrates 1994). Similarly, the band at 800 cm^−1^ was ascribed to Si-C and unsaturated esters including aromatic. These findings suggest that MB can adsorb on SS-Cl via aromatic rings that does not affect vibration of C=S bond. In case of SS-NO_3_, no bands, which could be directly related to MB, were found. The initial carbonate bands vanished, while a broad band of various sp^2^ and sp^3^ hydrocarbon bonds intensified. A narrow band at 875 cm^−1^ was ascribed to double bonds C=O and C=C, while a wide band at 1024 cm^−1^ can be associated with C-O– and Si-O–stretching vibrations. Furthermore, both samples showed stronger H_2_O bands after MB adsorption. There is a remarkable change of the intensities of infrared bands with no change positions. These results indicated a preferential involvement of certain functional groups in the adsorption of dye ions on the surface of the treated samples.

The findings of BET surface area, pore volume, and average pore diameter from various studies of modified slag materials and a selected number of commercial adsorbents were also summarized in Table 2. Upon comparison with the literature of modified slag-based materials, it is revealed that the as-prepared material SS-Cl showed the highest surface area, outperforming other studies reported earlier, even many that also use the same reagents (dissolution and coprecipitation treatment: HCl–NaOH). Only, the study of Gao et al. (2017) and Kuwahara et al. (2010) reported similar or highest BET area results respectively. However, this enhanced result require a separation of the residual silica from BFS via a hydrothermal treatment and the use of additional reagent NaAlO_2_, resulting in production yield decreased and a cost increased. Besides this, it shows that the reported values of surface area on commercial adsorbents are in general larger that the reported values of modified slag-based materials, but the as-prepared SS-Cl and SS-NO_3_ are in the same order. Furthermore, the synthetized samples show larger pore diameters than the reported values of commercial adsorbents. SS-Cl and SS-NO_3_ samples show values only comparable with the silica gel–large pore.

**Table 2:** Comparative of BET surface area, pore volume and average pores diameter data of the analyzed samples with the literature data of various modified slag materials and of a selected number of commercial adsorbents (na, data not available)

Material		BET area (m^2^/g)	Pore volume (cm^3^/g)	Average pore diameter (nm)	Reference
Samples in this study	SSS	3.47	0.02	20.26	This work
	SS-Cl	232.11	0.82	14.61	This work
	SS-NO_3_	110.07	0.25	9.04	This work
	SS-SO_4_	1.97	0.01	29.49	This work
	AC	813	0.46	2.26	This work
	BFS (dissolution–coprecipitation; H_3_PO_4_-NaOH)	97.8–164.6	0.2–0.216	na	(Kuwahara et al. 2009)
	BFS (HCl treatment)	0.90	na	na	(Xue et al. 2009)
Modified slag	BFS (dissolution–coprecipitation; HCl-NaOH)	11.9–23.5	na	na	(Kuwahara et al. 2010)
	BFS (dissolution–coprecipitacion HCl-NaOH+NaAlO_2_)	590–740	0.23–0-29	na	(Kuwahara et al. 2010)
	BFS dissolution–coprecipitation; HCl-NaOH)	245	0.180	3.5	(Gao et al. 2017)
	BFS (dissolution–coprecipitation; HCl-NaOH)	154	0.62	na	(Kuwahara et al. 2013)
	BFS (dissolution–coprecipitation; HCl-NaOH)	219	0.78	na	(Kuwahara et al. 2013)
	**Thermal treatment**	**8.62**	**112,000**	**5.58**	(Yu et al. 2015)
	Mechanical treatment	9.53	0.025	na	(Yang et al. 2018)
	Salicylic acid–methanol modification	66.655	0.153	3.832	(Cheng et al. 2018)
	BFS (dissolution–hydrothermal process; acid formic)	128	0.388	12.3	(Kuwahara et al. 2020)
Commercial adsorbents	Activated alumina	320	na	1.0–7.5	(Buekens and Zyaykina 2001)
	Silica gel:				
	Small pore	750–850	na	2.2–2.6	(Buekens and Zyaykina 2001)
	Large pore	300–350	na	10–15	(Buekens and Zyaykina 2001)
	Activated carbon:				
	Small pore	400–1200	na	1.0–2.5	(Buekens and Zyaykina 2001)
	Large pore	200–600	na	> 3	(Buekens and Zyarykina 2001)
	Molecular sieve carbon	400	na	0.2–1.0	(Buekens and Zyaykina 2001)
	Molecular sieve zeolites	600–700	na	0.3–1.0	(Buekens and Zyaykina 2001)
	Polymeric adsorbent	80–700	na	4.0–2.5	(Buekens and Zyaykina 2001)

Table 3 summarizes the findings from various studies on the adsorption capacities of slag-based materials of various dyes in water. It should be noted that the most of the previous works using modified slag materials described in Table 2 were focused fundamentally on the removal in water of phosphate (Kuwahara et al. 2010; Kuwahara et al. 2013; Yu et al. 2015), metal ions (Kuwahara et al. 2013), and polycyclic aromatic hydrocarbons (Yang et al. 2018), or VOCs (Kuwahara et al. 2009), and CO_2_ capture in air (Kuwahara et al. 2020). Only Cheng et al. (2018), Gao et al. (2017), Xue et al. (2009) elucidated the efficacy of adsorption of dyes in water by slag-based materials. However, the adsorption data proposed by these authors and the data reported in this study vary greatly, possibly compounded by the heterogeneous nature of the slags, and the different dyes and experimental conditions used (see Table 3). Thus, the interpretation and comparison of adsorption capacities between the studies are complicated.

**Table 3:** Comparative of reported maximum adsorption capacities of dyes and efficiencies of the analyzed samples and various modified slag materials (*m*, mass of adsorbent; *v*, volume of dye solution)

Material		Experimental conditions		Adsorption capacity (mg/g)	Adsorption efficiency (%)	Reference
Samples in this study	SSS	Dye: methylene blue; Co = 30 mg/l pH = 8; T° = 293 K; m/v = 3 g/l	Time = 240	7.17	74	This work
	SS-Cl		Time = 150 h	9.35	96	This work
	SS-NO_3_		Time = 240 h	8.97	93	This work
	SS-SO_4_		Time = 240 h	0	0	This work
	AC		Time = 24 h	10	100	This work
Modified slag	BFS (HCl treatment)	Co = 25–500 mg/l pH = 2; T° = 293 K; m/v = 5 g/l; time = 3 h	Dye:reactive blue 19	60	na	(Xue et al. 2009)
			Dye: reactive black 5	76	na	
			Dye: reactive red 120	55	na	
	Salicylic acid–methanol modification	Dye: methylene blue		41.62	42	(Cheng et al. 2018)
		Co = 200 mg/l				
		pH = 7				
		T° = 293 K				
		m/v = 2 g/l				
		Time = 3 h				
	BFS dissolution–coprecipitation; HCl-NaOH	Dye: methyl orange		167	99	(Gao et al. 2017)
		Co = 25 mg/l				
		pH = 7				
		T° = 293 K				
		m/v = 2.5 g/l				
		Time = 25 min				

In spite of these comparative difficulties, the maximum absorption capability of this study, that corresponding with SS-Cl (9.35 mg/g) is lower than the data reported by Cheng et al. (2018), and it requires more time to achieve the equilibrium. Despite both studies using similar experimental conditions and the same dye, the efficiency of our modified slag material, whose BET surface area is more than three time greater, and seems that can be limited by the MB amount used in the experiments (almost the entire dye solution was adsorbed, 96%). The adsorption efficiency is to be highly dependent on the initial dye concentration. The percentage adsorption decrease with increase in initial dye concentration but the actual amount of dye adsorbed per unit mass of adsorbent increased with increase in dye concentration in the test solution.

Nevertheless, the various studies highlighted so far, and the present study using stainless steel slags for the first time, have converged on the technical merit of slags can be used as adsorbents for remediating industrial effluents and wastewaters. Besides this, the obtained results remark that the studied conversion processes may enable the fabrication of potential adsorbents through low complex chemical components, at low cost and abundant supply, which would meet the requirement for mass production. The USGS-MRP (United States Geological Survey—Mineral Resources Program) stablished that the selling prices in 2015 of steel slag ranged from $1.65 to $36.93 per metric ton, with an average of $5.49 per metric ton. The market for slag is likely to fluctuate, but the government and industry efforts to promote “sustainable” materials and practices and recycling in general likely will favor the increased use of slags and it cost lowering. Furthermore, the chemicals used in this study for the transformation to adsorbent can be cheaply provided by the industrial supplies, without the use of costly calcium- or silicon-containing chemical or multiple pure metal-containing chemical sources. Comparison with the other adsorbents such as activated alumina (US$ 0.60–1.19 × 10^3^ t^−1^) (Liu et al. 2010), carbon nanotubes (US$ 441.85 × 10^6^ t^−1^), modified graphene oxide (about US$ 60 × 10^4^ t^−1^), zeolite (US$ 30–120 × 10^3^ t^−1^) (Rafatullah et al. 2010), and coconut shell–based activated carbon (US$ 0.88–1.32 × 10^3^ t^−1^) (Liu et al. 2010), the cost of modified SSS (SS-Cl and SS-NO_3_) will surely be much lower. The versatility the described synthetic methods is also enhanced by the fact that a settling process can easily separate the adsorbent and the liquid after the treatment, which enables the reuse of the adsorbent due to its high density.

## Conclusions

A three acid-base conversion process on raw stainless steel slags (SSS) to obtain low-cost adsorbents was proven using HCl, HNO_3_, and H_2_SO_4_ and subsequently NaOH reported earlier. A porous silica matrix with a mean pore diameter of 14.61 nm (in the range 0.6–300 nm) and a large surface area BET of 232.11 m^2^/g was successfully synthesized using HCl (SS-Cl) as a dissolving agent, while the use of HNO_3_ (SS-NO_3_) resulted in the formation of calcium silicate materials with a pore diameter of 9.04 mm and 110.07 m^2^/g. In contrast, the treatment with H_2_SO_4_ generated a low-surface area material (1.97 m^2/^g), even lower than the raw SSS (3.47 m^2^/g). SS-Cl and SS-NO_3_ samples exhibited good adsorption performance (9.35 mg and 8.97 mg/g respectively) for removing methylene blue (MB) from water. Even though the time required for adsorption was much longer, both samples exhibited adsorption capabilities comparable to the activated carbon used as a reference (9.72 mg/g). The enhanced adsorption capability of these samples is related to the improvement of BET. The obtained results demonstrated that SSS waste material can be valorized by conversion into a low-cost adsorbent. Although more detailed studies on process optimization, repeatability, and reproducibility of the process and environmental assessment need to be done before practical applications, the conversion processes could be considered to be beneficial from the viewpoint of realizing an effective use of SSS, and will offer novel solutions not only to waste management problems but to environmental problems, since the synthesized product can be used as a versatile adsorbent.
